# Indigenous knowledge for plant species diversity: a case study of wild plants' folk names used by the Mongolians in Ejina desert area, Inner Mongolia, P. R. China

**DOI:** 10.1186/1746-4269-4-2

**Published:** 2008-01-16

**Authors:** Khasbagan, Soyolt

**Affiliations:** 1 Institute of Ethnobotany, Inner Mongolia Normal University, Huhhot, 010022, Inner Mongolia, P.R. China

## Abstract

Folk names of plants are the roots of traditional plant biodiversity knowledge. This paper mainly records and analyses the wild plant folk names of the Mongolians in the Ejina desert area based on a field survey for collection and identification of voucher specimens. The results show that a total of 121 folk names of local plants have correspondence with 93 scientific species which belong to 26 families and 70 genera. The correspondence between plants' Mongol folk names and scientific species may be classified as one to one correspondence, multitude to one correspondence and one to multitude correspondence. The Ejina Mongolian plant folk names were formed on the basis of observations and an understanding of the wild plants growing in their desert environment. The high correspondence between folk names and scientific names shows the scientific meaning of folk botanical nomenclature and classification. It is very useful to take an inventory of biodiversity, especially among the rapid rural appraisal (RRA) in studying biodiversity at the community level.

## Background

Indigenous knowledge is the systematic information that remains in diverse social structures. It is usually unwritten and preserved only through oral tradition, and it refers to the knowledge system of indigenous people and minority cultures. Traditional knowledge of biodiversity concerns the names, uses, and management of plants and animals as perceived by the local and or indigenous people of a given area. Folk names of plants and animals are the roots of traditional biodiversity knowledge. Berlin has indicated a strong need for linking the scientific and folk systems of classification [1]. Examples of such links have been quoted by Berlin et al. who has looked at the relationship between folk names and scientific names [1-4]. For this reason, it has been brought into "Convention on Biological Diversity" (CBD). Precisely, in article 8 of CBD which describes that "subject to its national legislation, respect, preserve and maintain knowledge, innovations and practices of indigenous and local communities embodying traditional lifestyles relevant for the conservation and sustainable use of biological diversity and promote their wider application with the approval and involvement of the holders of such knowledge, innovations and practices and encourage the equitable sharing of the benefits arising from the utilization of such knowledge, innovations and practices". Besides, folk systems of naming and classification transmitted from generation to generation, is constantly recreated by communities and groups in response to their environment. In this case, it undoubtedly fell into the category of "Intangible Cultural Heritage" (ICH) of humanity. In "Convention for the Safeguarding of the Intangible Cultural Heritage", "oral traditions and expressions, including language as a vehicle of the intangible cultural heritage" and "knowledge and practices concerning nature and the universe" were domains of the ICH.

In pace with social change and development, the Mongols are changing from nomadic people into settlement residents. The knowledge concerning grassland ecosystems is vanishing gradually because the related knowledge is no longer useful to the Mongols who are settled down or engaged in farming or other economic pursuits. The Mongolians in Inner Mongolia have been influenced by the Chinese culture, e.g. in some areas Han Chinese words, including plant names, are more or less mixed up with the Inner Mongolians' spoken language. This may be leading to the Mongols forgetting traditional botanical knowledge related to the language of plant folk names and classifications.

Both artificial and natural factors lead to degradation of the grassland and desertification. As a result, plant diversity that Mongolians traditionally named and used has decreased. The reduction of plant diversity may also lead to the extinction of the related knowledge of biodiversity. Thus it will be impossible to hand down to future generations. For this reason, collection and analysis of plant folk names of the Mongolians is extremely important.

Ethnobotanical studies in Inner Mongolia have been carried out since the 1980s, having studied useful plants of herdsmen [5], folk nomenclature [6,7]. However, ethnobotanical findings are still preliminary and fragmentary. Particularly, indigenous Mongolian traditional knowledge of desert plant diversity has been neglected by biologists and anthropologists. Biodiversity has social, economic, ecological and ethical value. Understanding ecological functions of biodiversity, respecting the ethics and social importance of biodiversity, and the appropriate exploitation and use of biodiversity are the global issues facing biodiversity today. Scientists have paid close attention to the relationships between biodiversity and cultural diversity [8-11].

Mongolian traditional knowledge of biodiversity includes aspects of folk nomenclature, and traditional use and management of regional biodiversity. In this paper, in accordance with the ethnobotanical collections of the Mongolian folk names of wild plants in the Ejina desert area, the relationship between folk names and scientific names are studied, and the structure of Ejina Mongolian folk botanical nomenclature is also analyzed.

## Materials and methods

### Study area and ethnic group

The Ejina desert area is located in western Inner Mongolia of China, at 39°52'20" ~ 42°47'20"N and 97°10'23" ~ 103°7'15"E, with a land area of 102461.30 km^2^, the altitude ranges from 820 m to 1400 m. This area has a temperate zone continental climate, with an average annual temperature of 8.3°C, and a mean rainfall of only 38.2 mm. The frost-free period in the area is 145 days [12]. The Ejina desert area is part of the Alashan desert, which is a part of the Middle Asian desert region of the Asia African Desert region. According to the flora regional system of Inner Mongolia, the area belongs to Typical Desert Zone of the Warm-Temperate Desert Zone [13,14]. The main landscape of this area is desert, which includes sandland, oasis, gobi and lower mountain-monadnock rocky desert etc.

According to related materials [13-16], and our investigations, nearly 200 species of vascular plants are distributed in this area. Scientists have attached importance to the biodiversity of the Ejina desert area. Biological and ecological research projects have been carried out in the area. However, previous research mainly focused on the natural ecology [17-21]. Few scientists paid attention to ethnobiological problems of the interrelationship between local people and biodiversity.

Local peoples in this area are the Torgod Mongolians, one of the descendent tribes of the Mongolian nationality. From 1731 A. D. they have occupied this area and engaged in a traditional nomadic livelihood. At present there are more than 5000 Mongolians in this area.

## Methods

During 2001 to 2005, the authors have been to the Ejina Banner 6 times. Field work was done in 12 villages and 55 local Mongol herdsmen (informants) were interviewed. Methods of ethnobotanical interviewing, including key informant interviews, free-listing, and open-ended questionnaires were used. After going to each village, the authors identified elder herdsmen's families and paid a formal visit. Mongolian oral language was used as the working language and findings were originally recorded in Mongolian written language. Scientific names of plants are defined through collection and identification of voucher specimens.

## Results and discussion

A total of 119 folk names of local plants are recorded. Based on the results of identifying the specimens, the folk names corresponded with 91 scientific species which belong to 26 families and 70 genera. The rate of Correspondence was 76.47% between folk Mongol names and scientific names (table 1).

**Table 1:** The Correspondence between folk names of the Mongolians in Ejina desert area and scientific classification

**Family**	**Folk names**	**Scientific names**
Apocynaceae	**olus**	*Poacynum pictum *(Schrenk) Baill.
Asclepiadaceae	**hucu**	*Cynanchum cathayense *Tsiang et Zhang
Boraginaceae	**dumug**	*Arnebia guttata *Bunge
	**begesun zhanggu; nas wugai ebes**	*Lappula deserticola *C. J. Wang
Chenopodiaceae	**sulker**	*Agriophyllum pungens *(Vahl) Link ex A. Dietr.
	**noosut; noosut hamhag**	*Bassia dasyphylla *(Fisch. et Mey.) O. Kuntze
	**temljgen; noil**	*Chenopodium acuminatum *Willd.
	**noil**	*Chenopodium album *L.
	**noil**	*Chenopodium glaucum *L.
	**cagan keres; kerest hamhag**	*Corispermum mongolicum *Iljin.
	**keres**	*Halogeton glomeratus *(Bieb.) C. A. Mey.
	**zag; sagleger zag; xikur zag; yabgan zag**	*Haloxylon ammodendron *(C. A. Mey.) Bunge
	**xira kureg; kureg**	*Kalidium foliatum *(Pall.) Moq.
	**kureg**	*Kalidium sinicum *(A. J. Li) H. C. Fu et Z. Y. Chu
	**har keres; narin cagan**	*Kochia scoparia *(L.) Schrad.*var. sieversiana *(Pall.) Ulbr.
	**hushit**	*Micropeplis arachnoidea *(Moq.) Bunge
	**bor budrgen**	*Salsola passerina *Bunge
	**baglur**	*Salsola pestifer *A. Nelson
	**wurgest**	*Salsola pellucida *Litv.
	**cagan but**	*Sympegma regelii *Bunge
Compositae	**wunurt xiaralj**	*Artemisia caespitosa *Ledeb.
	**cagan xiabag; agi**	*Artemisia dalai-lamae *Krasch.
	**xiaralj; agi**	*Artemisia frigida *Willd.
	**xiar xiabag; tatenghai**	*Artemisia songarica *Schrenk.
	**xiar mod**	*Asterothamnus centrali-asiaticus *Novopokr.
	**honggurzul**	*Cirsium arvense *(L.) Scop.
	**kuji ebes**	*Inula salsoloides *(Turcz.) Osteuf.
	**sutai nogo**	*Ixeris chinensis *(Thunb.) Nakai
	**dalen tobqi**	*Karelinia caspia *(Pall.) Less.
	**gaxiun**	*Serratula centauroides *L.
	**gaxiun ebes**	*Sonchus arvensis *L.
	**sutai nabqi**	*Taraxacum leucanthum *(Ledeb.) Ledeb.
Convolvulaceae	**oryamug**	*Convolvulus arvensis *L.
	**cagan tolgait; elkendeg**	*Cardaria pubescens *(C. A. Mey.) Jarm.
Cruciferae	**lalajing**	*Lepidium obtusum *Basin.
	**shaga**	*Pugionium cornutum *(L.) Gaertn.
Cynomoriaceae	**wulan goyo; sozhung**	*Cynomorium songaricum *Rupr.
Cyperaceae	**xirki**	*Eleocharis mitracarpa *Steud.
	**xirki **	*Scirpus strobilinus *Roxb.
Elaeagnaceae	**jigd**	*Elaeagnus angustifolia *L.
Ephedraceae	**zergen**	Ephedra przewalskii Stapf
Frankeniaceae	**kureng ebes**	*Frankenia pulverulenta *L.
Gramineae	**tongge; deres**	*Achnatherum splendens *(Trin.) Nevski
	**ilbar**	*Aristida adscenionis *L.
	**cagan ebes**	*Cleistogenes squarrosa *(Trin.) Keng
	**budnur; hazaar ebes**	*Crypsis aculeata *(L.) Ait.
	**kag**	*Leymus secalinus *(Georgi) Tzvel.
	**hulus; acamag; shagxig hulus; shaorag hulus; hanan hulus;ajirgan hana**	*Phragmites australi*s (Cav.) Trin. ex Steud.
	**suli**	*Psammochloa villosa *(Trin.) Bor.
	**narin ebes**	*Puccinellia hauptiana *(Trin.) Krecz.
Iridaceae	**cakildag; cakirma**	*Iris lactea *Pall.*var. chinensis *(Fisch.) Koidz.
Lguminosae	**cegereg**	*Alhagi maurorum *Medic.*var. sparsifolium *(Shap.) Yakovl.
	**munk hargen**	*Ammopiptanthus mongolicus *(Maxim.) Cheng f.
	**hatinggir**	*Astragalus hamiensis *S. B. Ho
	**alten hargen**	*Caragana leucophloea *Pojark.
	**xiker buyaa**	*Glycyrrhiza uralensis *Fisch.
	**zara wurges; ortud**	*Oxytropis aciphylla *Ledeb.
	**hor;tom; sogtu ebes**	*Oxytropis galbra *(Lam.) DC.
	**horen buyaa**	*Sophora alopecuroides *L.
	**porqigenur; honht ebes**	*Sphaerophysa salsula *(Pall.) DC.
Liliaceae	**humel**	*Allium mongolicum *Regel
	**taan**	*Allium polyrhizum *Turcz. ex Regel
Orobanchaceae	**cagan goyoo**	*Cistanche deserticola *Ma
	**budergen in goyoo; zag in wug**	*Cistanche sinensis *G. Beck
Plumbaginaceae	**zer in deleng**	*Limonium aureum *(L.) Hill ex O. Kuntze
	**zer in deleng**	*Limonium bicolor *(Bunge) O. Kuntze
	**zer in deleng**	*Limonium tenellum *(Turcz.) O. Kuntze
Polygonaceae	**torleg**	*Calligonum gobicum *(Bunge) A. Los.
	**torleg; but**	*Calligonum mongolicum *Turcz.
	**wuyet wulan**	*Polygonum aviculare *L.
	**bajun**	*Rheum nanum *Siewers
	**kureng keres**	*Rumex marschallianus *Rchb.
Ranunculaceae	**moren hucu**	*Clematis orientalis *L.
Rosaceae	**hatu har; boiles**	*Prunus mongolica *Maxim.
Salicaceae	**torai; nailjag; zulzagan torai**	*Populus euphratica *Oliv.
	**burgas**	*Salix cheilophila *Schneid.
Scrophulariaceae	**zer in del**	*Dodartia orientalis *L.
Solanaceae	**nohai harmeg ; wurgest harmeg**	*Lycium ruthenicum *Murr.
Tamaricaceae	**wulan budergen; budergen**	*Reaumuria soongarica *(Pall.) Maxim.
	**yamaan suhai**	*Tamarix leptostachys *Bunge
	**wulan suhai**	*Tamarix ramosissima *Ledeb.
Zygophyllaceae	**wuseg**	*Nitraria roborowskii *Kom.
	**tosun harmeg; cagan harmeg**	*Nitraria sibirica *Pall.
	**temer wuseg; wuseg**	*Nitraria sphaerocarpa *Maxim.
	**wusun wuseg**	*Nitraria tangutorum *Robr.
	**wumkei ebes**	*Peganum harmala *L.
	**yamaan zhanggu**	*Tribulus terrestris *L.
	**botgen tabeg; hulen nai wumdan**	*Zygophyllum fabago *L.
	**argal in wumd**	*Zygophyllum gobicum *Maxim.
	**wulen nai botgen tabeg**	*Zygophyllum loczyi *Kanitz
	**bojignur; nohai xiereng**	*Zygophyllum xanthoxylum *(Bunge) Maxim.

### The correspondence between plant folk names & scientific names

The plants folk names and scientific names (species) are not a simply one to one correspondence. It may be organized as below:

(a) One to one correspondence One folk name has correspondence with one scientific species. For example, the folk name **sulker **only corresponds with *Agriophyllum pungens***, zhergen **with *Ephedra przewalskii*, **jigd **with *Elaeagnus angustifolia*, **chegereg **with *Alhagi maurorum *var. *sparsifolium*, **suli **with *Psammochloa villosa*, **baglur **with *Salsola pestifer*, **humel **with *Allium mongolicum*, **taan **with *Allium polyrhizum *and **olus***Poacynum pictum *etc.

(b) Multitude to one correspondence Two or more folk names have correspondence with only one scientific species. For example, **bor bodurgen **and **xira huhurgene **correspond with *Kalidium foliatum*, **wulan goyo **and **sozhung **with *Cynomoricum songaricum*; **zag, sagleger zag, xikur zag, yabgan zag **correspondence with *Haloxylon ammodendron*; **hulus, acamag, shagxig hulus, shaorag hulus, hanan hulus, ajirgan hana **correspondence with *Phragmites australis *etc. In this case, those folk names correspondence with one scientific name are regarded as a folk synonym.

(c) One to multitude correspondence One folk name corresponds with two or more scientific species. For example, **zherin deleng **corresponds with *Limonium aureum*, *Limonium tenellum *and *Limonium bicolor*, **noil **with *Chenopodium acuminatum and Chenopodium album*, **xirki **with *Eleocharis mitracarpa *and *Scirpus strobilinus *etc. In this case, those folk names with correspondence with two or more scientific names are regarded as folk homonyms.

### Structure of Ejina Mongolian Folk Botanical Nomenclature

A basic step in analyzing the structure of folk botanical nomenclature is to tell the difference between primary and secondary names and to distinguish between the various primary names [22]. According to the result of the Mongolian linguistic analysis, the Mongolian folk names of wild plants in the Ejina desert area are distinguished as primary names and secondary names.

#### Primary names

A primary name is considered to be 'semantically unitary' which means that it is a single expression, even if composed of more than one constituent. Many primary names have just a single constituent, and they belong to simple primary names, such as **bodurgan, bogqignuur, boya, chaheldeg, chaherma, chegereg, deres, goyo, hamhag, hargan, harmag, hatingir, heres, hucu, huhurgen, hulusu, humul, jigd, kag, olos, soli, sozhung, suhai, taan, torai, torlog, tunge, tunghul, wusug, xirki, zhergen **etc. In the Mongolian language, these words are proper names which haven't other meanings. Other primary names are composed of more than one constituent which belongs to complex primary names. Complex primary names consist of two Mongol words. Some complex primary names include a word such as **bot **[shrub] or **ebes **[grass] which indicates the life form, such as **chagan bot, chagan ebes, honht ebes, huji ebes, narin ebes, sogtuu ebes, wumhei ebes **etc. In this type of folk classification, a word **bot **or **ebes **serves as a taxon such as family or genus in scientific taxonomy. These types of names belong to productive complex primary names. Other complex primary names don't include a word to express a folk taxon, belonging to the productive complex primary name, such as **botgon tabag, dalan tobqi, hulnai wumdagan, wusun hor, wuyet wulan, zherin deleng **etc.

#### Secondary names

Secondary names are formed from simple primary names by simply adding a modifier which further describes the plant. Among these types of names, simple primary names serve as a folk generic. For example, secondary names **wulan suhai **(*Tamarix ramosissima*) and **imaan suhai **(*Tamarix leptostachys*) are formed from the simple primary name **suhai**, and **xihir boya **(*Glycyrrhiza uralensis*) and **horen boya **(*Sophora alopecuroides*) are formed from **boya**. A word **suhai **serves as a folk generic and equals to the scientific genus *Tamarix *(Tamaricaceae). But a folk generic **boya **usually is used to name the plants which have fleshy roots, and it isn't specially appointed to a scientific genus. Among the plant folk names in the Ejina desert area, there are 10 folk generic names collected. The relationship between folk specific, folk generic and scientific species and family names can be seen in table 2.

**Table 2:** Relationship between folk specific, folk generic and scientific species and family

**Folk generic**	**Folk specific**	**Scientific species**	**Scientific family**
**bodurgan**	**bor bodurgen**	*Kalidium foliatum*	Chenopodiaceae
	**wulan bodurgan**	*Reaumuria soongarica*	Tamaricaceae
**boya**	**horen boya**	*Sophora alopecuroides*	Leguminosae
	**xihir boya**	*Glycyrrhiza uralensis*	Leguminosae
**goyo**	**chagan goyo**	*Cistanche sinensis*	Orobanchaceae
		*Cistanche deserticola*	
	**wulan goyo**	*Cynomoricum songaricum*	Cynomoriaceae
**hamhag**	**herest hamhag,**	*Corispermum mongolicum*	Chenopodiaceae
	**noosun hamhag**	*Bassia dasyphylla*	Chenopodiaceae
**hargan**	**altan hargan**	*Caragana leucophloea*	Leguminosae
	**munh hargan**	*Ammopiptanthus mongolicus*	Leguminosae
**harmag**	**chagan harmag**	*Nitraria sibirica*	Zygophyllaceae
	**nohai harmag**	*Lycium ruthenicum*	Solanaceae
**heres**	**heres**	*Halogeton glomeratus*	Chenopodiaceae
	**chgan heres**	*Corispermum mongolicum*	Chenopodiaceae
	**har heres**	*Kochia scoparia *var.* sieversiana*	Chenopodiaceae
**hucu**	**hucu **	*Cynanchum cathayense*	Asclepiadaceae
	**morin hucu**	*Convolvulus arvensis*	Convolvulaceae
**suhai **	**imaan suhai **	*Tamarix leptostachys*	Tamaricaceae
	**wulan suhai**	*Tamarix ramosissima*	Tamaricaceae
**xiralji**	**xiralji **	*Artemisia songarica*	Compositae
	**wunurt xiralji**	*Artemisia caespitosa*	Compositae

## Discussion

The high correspondence between folk names and scientific names shows the scientific meaning of folk botanical nomenclature and classification. Ejina Mongolians' folk botanical nomenclature and classification is an important part of their natural culture. This type of knowledge and culture has a great effect on their adaptation to the desert environment, utilization of plant resources and traditional biodiversity management on the community level. The collection and analysis of plant and animal folk names is very useful to the inventory of biodiversity, especially among the rapid rural appraisal (RRA) in studying biodiversity at the community level. Sometimes the folk names lead to finding new species records in a given area. In this study the plant folk name **burgas **was recorded in advance and based on the descriptions of the plant's characteristics and habitats specimens were collected afterwards by local herdsmen. The result of the identification of specimens shows that the scientific name of **burgas **is *Salix cheilophila *Schneid. Thus a folk name led to finding this species in this area for the first time.

## Authors' contributions

The field work for data collection and analysis were conducted by all authors. Manuscript preparation was by Khasbagan. All authors read and approved the final manuscript.
